# Rotten to the Cortex: Ceramide-Mediated Lipotoxicity in Diabetic Kidney Disease

**DOI:** 10.3389/fendo.2020.622692

**Published:** 2021-01-28

**Authors:** Rebekah J. Nicholson, Marcus G. Pezzolesi, Scott A. Summers

**Affiliations:** ^1^Department of Nutrition and Integrative Physiology, University of Utah, Salt Lake City, UT, United States; ^2^Diabetes and Metabolism Research Center, University of Utah School of Medicine, Salt Lake City, UT, United States; ^3^Division of Nephrology and Hypertension, Department of Internal Medicine, University of Utah, Salt Lake City, UT, United States

**Keywords:** ceramide, sphingolipids, diabetic kidney disease, diabetic nephropathy, lipid metabolism, lipotoxicity

## Abstract

Diabetic kidney disease (DKD) is a prevalent and progressive comorbidity of diabetes mellitus that increases one’s risk of developing renal failure. Progress toward development of better DKD therapeutics is limited by an incomplete understanding of forces driving and connecting the various features of DKD, which include renal steatosis, fibrosis, and microvascular dysfunction. Herein we review the literature supporting roles for bioactive ceramides as inducers of local and systemic DKD pathology. In rodent models of DKD, renal ceramides are elevated, and genetic and pharmacological ceramide-lowering interventions improve kidney function and ameliorate DKD histopathology. In humans, circulating sphingolipid profiles distinguish human DKD patients from diabetic controls. These studies highlight the potential for ceramide to serve as a central and therapeutically tractable lipid mediator of DKD.

## Introduction

Diabetes is the global leading cause of chronic kidney disease (CKD) and end-stage renal disease (ESRD) (1, 2). An estimated 30–40% of patients with type 1 and type 2 diabetes develop CKD throughout the course of their disease (3, 4). Improvements over the last decades in the treatment of diabetes to improve blood glucose control (i.e. continuous blood glucose monitoring) have led to a general decrease in diabetes-related complications. However, kidney disease and failure have persisted as primary drivers of excess morbidity and mortality in diabetic patients (5, 6). Importantly, increases in diabetes and obesity prevalence are responsible for the projected increase in all-cause ESRD incidence in the United States (7).

Diabetic kidney disease (DKD) is thought to result from a progression of systemic and local insults, clinically represented by impaired renal function with or without elevated urinary albumin excretion (8, 9). Current DKD prevention and treatment strategies primarily target glycemic and hypertensive control; however, these therapeutic options are only capable of modest delays in disease progression toward renal failure (4). A deeper understanding of kidney-specific DKD disease mechanisms and development of more targeted therapeutics to meaningfully slow or halt disease progression are urgently needed. Investigations in human and animal studies have characterized glomerular and tubulointerstitial hypertrophy, inflammation, and sclerosis as key disease processes of DKD. Exciting evidence has qualified sphingolipids as potential drivers of renal pathology, with implicated roles in multiple features of DKD including altered lipid metabolism, insulin resistance, mitochondrial dysfunction, fibrosis, apoptosis, and vascular damage. The purpose of this review is to present relevant findings supporting the role of sphingolipids, with a focus on ceramide, in kidney-specific pathologies.

## Sphingolipid Metabolism

Sphingolipids make up a non-abundant, yet highly diverse, lipid class. The central metabolites of sphingolipid metabolism, ceramides, are produced *via* a *de novo* synthesis pathway in the endoplasmic reticulum (ER). Ceramides are subjected to further processing in the Golgi apparatus to produce complex sphingolipid molecules such as glycosphingolipids and sphingomyelins (SM). Importantly, dynamic breakdown and salvaging of select sphingolipids can also contribute to the re-synthesis of ceramides. As a whole, the sphingolipid pool influences cell membrane dynamics as well as cell growth, function, and programmed death (10). Studies manipulating enzymes involved in sphingolipid synthesis and metabolism continue to provide valuable information regarding the roles of sphingolipid flux and signaling in metabolism and disease.

*De novo* synthesis of ceramide begins in the endoplasmic reticulum with the condensation of an amino acid (i.e. L-serine) with an acyl-CoA (i.e. palmitoyl-CoA) to form a sphingoid backbone, a reaction that is catalyzed by serine palmitoyl transferase (SPT) (11). A family of six enzymes termed (dihydro)ceramide synthase (CERS1-6) adds a second, variable acyl group to sphinganine to form the dihydroceramides. The CERS enzymes are distinguishable by their substrate specificity and tissue distribution. CERS5 and 6 are upregulated by stress stimuli and promote the incorporation of long-chain (e.g. C16) acyl groups, which produce the deleterious C16-ceramides often implicated in metabolic dysfunction (12–19). Alternatively, CERS2 is more ubiquitously expressed, with particularly high expression in the kidney and liver. This enzyme produces very-long-chain (i.e. C20–26) ceramide species, which are considered benign or protective (20, 21). The fact that mammals and other species contain numerous, highly conserved CERS enzymes is noteworthy, and highlights the importance of the acyl chain length in ceramide signaling. As such, the CERS enzymes contribute significantly to the structural and functional diversity of sphingolipids (22). Regulatory activity of ceramides is also mediated by the addition of a double bond to the delta-4 carbon of the dihydroceramides by dihydroceramide desaturase (DES1 and 2). Animal studies reveal that genetic or pharmacological inhibition of DES1 reduces ceramides, induces accumulation of dihydroceramides, and attenuates metabolic dysfunction (23–25). This *de novo* ceramide synthesis pathway is upregulated in conditions of nutrient excess (i.e. oversupply of palmitate and serine) and in response to inflammatory agonists and glucocorticoids (26).

Ceramides can be further metabolized in the Golgi apparatus by a series of additional enzymes, which produce the complex species that make up the majority of the cellular sphingolipidome. For example, sphingomyelin synthases add a phosphocholine headgroup to ceramides, producing the most abundant sphingolipid class (i.e. the sphingomyelins) (27). Alternatively, glucosylceramide synthase adds a glucose moiety, producing the glucosylceramides (27). Glucosylceramides can receive additional carbohydrates through a series of enzymes that produce the complex ganglioside family. Ceramides can also be deacylated by a family of ceramidases, which produce sphingosine. Curiously, the beneficial adipokine adiponectin activates a ceramidase activity within adiponectin receptors (AdipoRs), improving metabolic homeostasis and blocking apoptosis by degrading ceramides (28). Both sphingosine and ceramide can be phosphorylated by specific kinases, which produce sphingosine-1-phosphate and ceramide-1-phosphate (29–31). These phosphorylated species have additional signaling functions, often opposing the actions of ceramides.

## Sphingolipids in DKD

DKD shares similar risk factors and disease mechanisms to diabetes, cardiovascular disease (CVD), obesity, and steatohepatitis, conditions associated with deregulation and accumulation of bioactive sphingolipids (14, 27). As such, considerable attention has turned to the role of sphingolipids in DKD.

Numerous studies have demonstrated that circulating sphingolipid profiles differ in humans with DKD *versus* diabetic controls. In subjects with type 1 diabetes recruited as part of the Diabetes Control and Complications Trial (DCCT) and long-term follow-up (EDIC) cohort, lower baseline levels of very-long-chain ceramides (C20–26:1) were associated with worsening albuminuria over 14–20 years (32). A second report by the same group noted distinct associations between specific glycosphingolipid species and development of macroalbuminuria and chronic kidney disease (33). A metabolomic screen in type 2 diabetes patients with early and overt DKD reported positive associations between C16 ceramide, C16 SM, C18 glucosylceramide, and sphingosine with urinary albumin-creatinine ratio (34). Notably, several reports have documented associations of SM and kidney disease in type 1 diabetes, wherein SM correlates positively with albuminuria (35), rapid estimated glomerular filtration rate (eGFR) decline, and progression toward ESRD (36). Conversely, Tofte and colleagues reported inverse associations of several SM species and longitudinal kidney disease endpoints including renal impairment, ESRD, and all-cause mortality (37).

Whether differences in serum sphingolipids are reflective of altered kidney sphingolipid metabolism or are attributable to changes in non-renal tissues remains unanswered by human studies. The one piece of supportive data from patients is by Eckes et al., who found that CERS5 and CERS6 were upregulated in kidney cortices of humans (and mice) with non-diabetic kidney fibrosis (38). Nonetheless, numerous studies have reported that kidney-specific changes in sphingolipids and/or sphingolipid-metabolizing enzymes occur in animal and cell models of DKD. For example, investigators have shown that enzymes controlling ceramide production are upregulated in kidneys from mouse models of DKD (e.g. the SPTLC2 subunit of the SPT complex in db/db mice) (39). Exposing cultured glomerular cells to elevated glucose, free fatty acids, and angiotensin II, which recapitulate the environment in diabetes, elevates ceramides (40) and hexosylceramides (41). Additionally, several studies have demonstrated sphingolipid accumulation in kidney cortices of C57BLKS *db/db* diabetic mice (41, 42) or rats with streptozotocin (STZ)-induced diabetes (43, 44). In *db/db* eNOS deficient mice, MALDI imaging mass spectrometry was utilized to visualize GM3 and sulfoglycosphingolipid accumulation in kidney glomeruli and tubules, respectively (45). Notably, Sas et al. reported ceramide depletion in kidney cortices of mice with more advanced DKD (39). This corresponds to recent work tracking increases in urine ceramides in human patients with early DKD (i.e. CKD stages 1–3), but not CKD stage 4, compared to diabetic and healthy controls (46). Lastly, the Huang group has built a body of work to demonstrate modulation of sphingosine kinase activity in regulating mesangial cell proliferation and extracellular matrix (ECM) deposition under conditions of elevated glucose or advanced glycation end products (AGEs) (47–49).

## Local and Systemic Insults That Drive DKD

DKD is clinically diagnosed by a persistent reduction in eGFR less than 60 ml/min per 1.73 m^2^ and/or the presence of a chronically high urinary albumin-to-creatinine ratio above 30 mg/g (4). However, analysis of DKD presentation between the time periods of 1988 to 1994 and 2009 to 2014 suggests that fewer DKD patients present with albuminuria; more manifest declining eGFR, and some develop histologically distinguishable DKD before presentation of clinical markers (50, 51). Both type 1 and type 2 diabetes are characterized by chronic hyperglycemia, which is a risk factor of DKD and often accompanied by other systemic perturbations, such as hypertension and obesity. Additionally, DKD is associated with marked hyperlipidemia with elevated levels of circulating LDL, oxidized-LDL, VLDL, triglyceride, and free-fatty acids (FFA) and low HDL in both type 1 and type 2 diabetes (8, 52).

**Figure 1:**
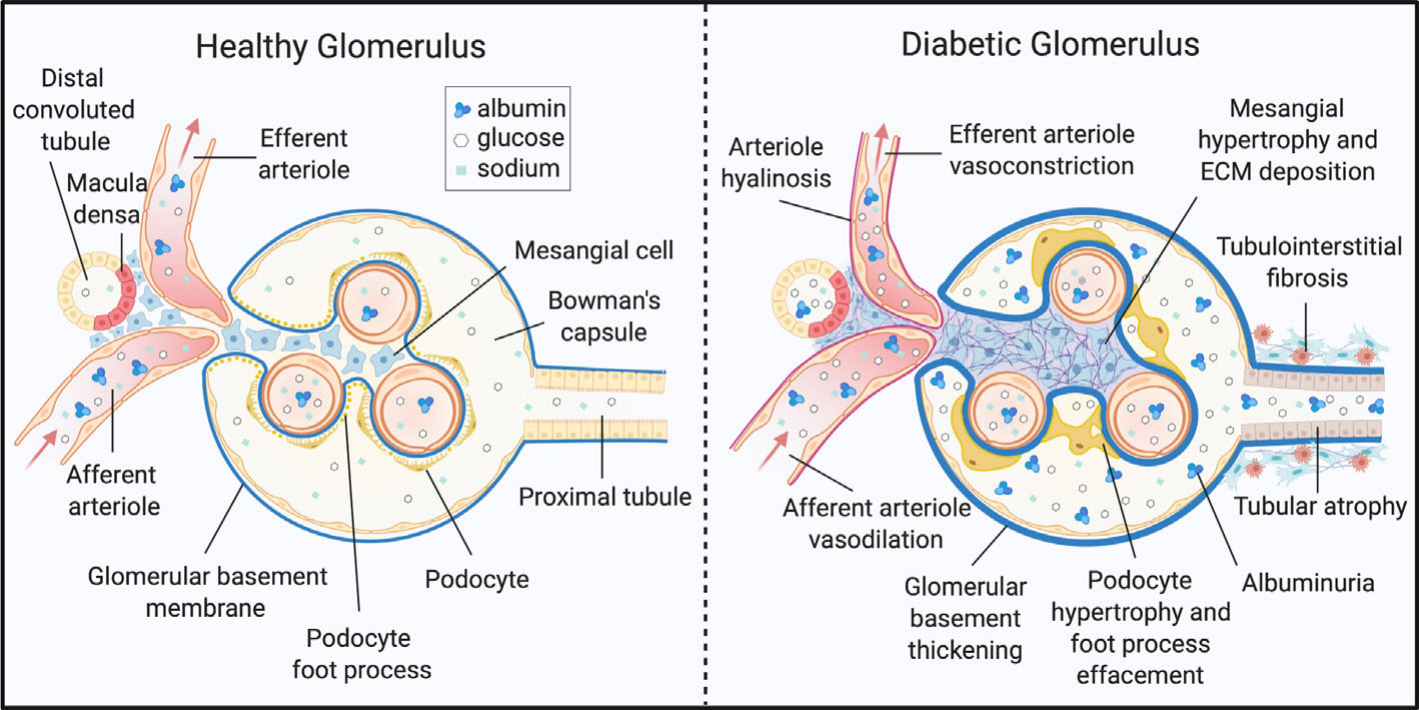
Histopathology of diabetic kidney disease. DKD elicits structural and functional changes to the glomerulus, tubules, and microvasculature of the kidney. ECM, extracellular matrix. Created with BioRender.com.

The kidney is a highly metabolic tissue and houses a multitude of terminally differentiated cell types. As such, developing a comprehensive understanding of DKD onset and progression in different cell types is a monumental challenge. Nevertheless, distinguishable metabolic and histopathological alterations of DKD have been documented, and some are well-described (**Figure 1**). Structurally, DKD is distinguishable by the early thickening of the glomerular basement membrane (GBM) (4, 8, 52, 53). Additionally, mesangial cells within the glomerulus undergo marked hypertrophy and fibrosis. Podocytes, cells essential for maintaining the size-restriction barrier of the glomerulus, experience foot process effacement, hypertrophy, and apoptosis. Diabetic kidneys are also defined by atrophy of the brush border within renal tubules, tubular epithelial cell (TEC) loss, and TEC dedifferentiation. Tubular and glomerular cells are susceptible to lipid accumulation, and lipid-rich lesions were noted in initial reports of DKD (54). The renal interstitium, defined as the ECM, fluid, and cells surrounding nephrons and capillaries, is also subject to pathological changes. Activation and migration of peripheral or local immune cells, as well as mesenchymal-TECs accumulate in DKD kidneys and induce inflammation and ECM deposition. Progression of DKD drives interstitial and tubular fibrosis, as well as glomerulosclerosis (4, 8, 52, 53).

Recently, attention has been paid to renal steatosis as a marker of lipotoxicity in the diabetic kidney. Healthy renal cells—particularly proximal TECs, which are responsible for the majority of active reabsorption of sodium, glucose, and other metabolites from blood filtrate—rely primarily on fatty acid oxidation for ATP production (55, 56). Furthermore, terminally differentiated podocytes and TECs have limited metabolic flexibility and are highly susceptible to ATP depletion (55). Lipid accumulation in renal cells stems from both increased lipid uptake and impaired fatty acid oxidation (57). Exposure of kidney cells to free fatty acids could increase with the onset of proteinuria, in which albumin-bound FFAs are present in kidney filtrate (58). Additionally, podocytes and TECs contain swollen mitochondria with poorly defined cristae which undergo rapid fission and fusion, yet are not recycled *via* mitophagy (56, 59). The kidney also exhibits exceptional rates of protein turnover and fractional synthesis (53, 60). As such, renal cells are vulnerable to greater endoplasmic reticulum (ER) stress in addition to onslaughts from mitochondrial reactive oxygen species (ROS) production, systemic pro-inflammatory cytokines, and aggregation of advanced glycation end products (53, 57).

Lastly, the diabetic kidney undergoes multiple hemodynamic shifts. Hypertension is both a potential precursor and product of kidney injury. In the diabetic kidney, increased delivery of glucose and reabsorption in the proximal tubule by the SGLT2 sodium-glucose co-transporter decreases sodium concentrations reaching the macula densa (8). The resulting chronic activation of the systemic and local renin-angiotensin-aldosterone system (RAAS) stimulates blood vessel vasoconstriction and sodium resorption to increase blood pressure and induce glomerular hyperfiltration (61, 62). Additionally, decreased production of nitric oxide production by eNOS, elevated VEGF-B signaling, and increased endothelin-1 (ET-1) production results in hemodynamic imbalance, exacerbating pro-inflammatory and pro-fibrotic signaling (61, 63, 64).

## Genetic and Pharmacologic Inhibition of Ceramide Accumulation Improves DKD Endpoints

The question of whether ceramide plays a causative role in DKD pathology has been partially addressed in preclinical investigations. Notably, whole-body ceramide-lowering interventions improve kidney outcomes (40, 42). Diabetic rats and high fat diet (HFD)-fed mice subjected to 4 weeks of treatment with myriocin, an SPT inhibitor, had lower body weight, fasting blood glucose, plasma insulin, and circulating free fatty acid levels compared to diabetic and obese controls (40). Myriocin lowered kidney cortex ceramide and attenuated diabetes- or HFD-induced proteinuria, mesangial matrix and GBM thickening, and podocytopathy. Oral administration of AdipoRon, an adiponectin mimetic, effectively lowered ceramide in kidney cortices of *db/db* mice and improved glomerular mesangial thickening, steatosis, sclerosis, and immune cell infiltration (42). Importantly, AdipoRon treatment reversed kidney histopathology without changing body weight or whole-body glucose metabolism. Similarly, prevention of ceramide accumulation by intraperitoneal administration of an acid sphingomyelinase (ASM) inhibitor in *db/db* mice prevented proteinuria and glomerular injury (65). Use of rapamycin, an mTOR inhibitor, to treat rats with STZ-induced diabetes ameliorated mesangial matrix thickening and glomerular cell death (43). Interestingly, rapamycin treatment decreased SPT expression and cortical ceramide and SM levels in diabetic animals. This observation supports the idea that mTOR influences sphingolipid synthesis by regulating SPT and ceramide synthases (66).

Few studies have reported kidney-specific sphingolipid-lowering interventions in the setting of DKD. *In vitro* models have demonstrated that myriocin and AdipoRon have protective actions, lowering ceramide and preventing mitochondrial ROS production and cell apoptosis (40, 42). Additionally, promoting the production of anti-apoptotic sphingosine-1-phosphate (S1P) in HEK-293 cells significantly decreased rates of ceramide- or TNFα-induced apoptosis (67). At the time of this review, Guangbi and colleagues are the first and only group to produce a kidney-targeted *in vivo* model to study manipulation of renal ceramide metabolism (68). Specifically, knockout of acid ceramidase in podocytes (*Asah1^fl/fl^/Podo^Cre^*) of healthy mice led to glomerular ceramide accumulation and a concomitant increase in albuminuria, podocyte foot process effacement, and glomerular permeability. Furthermore, depleting glomerular ceramides with the additional knockout of ASM (*Smpd1^−/−^ Asah^fl/fl^/Podo^Cre^*) normalized proteinuria and podocytopenia.

## Proposed Mechanisms by Which Ceramides Drive DKD

**Figure 2:**
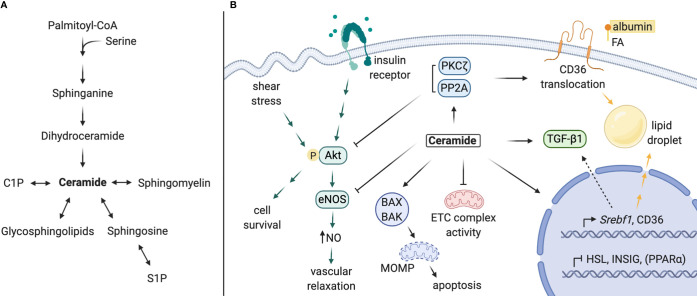
Proposed mechanisms of local ceramides in DKD pathology. **(A)** Schematic of sphingolipid metabolism. **(B)** Ceramides promote lipid accumulation *via* i) upregulation of CD36 and Srebf1 expression, ii) stimulation of CD36 translocation and SREBP-1 cleavage, and iii) inhibition of intracellular lipase expression. Ceramide and its targets PP2A and PKCζ inhibit Akt activation, impeding downstream cell survival (most cell types) or eNOS-dependent NO production (in endothelial cells). Ceramide antagonizes mitochondrial function by inhibiting ETC complex activity and permeabilizing mitochondrial membranes, leading to programmed cell death. Lastly, ceramide contributes to TGF-β signaling and tissue fibrosis, which may be partially mediated by SREBP-1. C1P, ceramide-1-phosphate; S1P, sphingosine-1-phosphate; FA, fatty acid; NO, nitric oxide; ETC, electron transport chain; MOMP, mitochondrial outer membrane permeabilization. Created with BioRender.com.

The role of ceramide as a lipotoxic mediator of metabolic dysfunction in CVD, diabetes, and fatty liver disease has been well-characterized (69). These conditions share common risk factors and disease processes with DKD. As such, we propose a comparable mechanism for ceramide-mediated pathology in kidney disease processes (**Figure 2**). Below, we detail various elements of a mechanism for ceramides in DKD:

### Lipid Accumulation

Transcriptomic analyses of kidney biopsies from human patients have revealed elevated expression of *SREBF* and *CD36* in the setting of DKD, which facilitate transcriptional upregulation of genes involved in *de novo* lipogenesis, triglyceride synthesis, and lipid uptake (13, 23, 70). Studies in other tissues (e.g. the liver) show that ceramide accumulation or depletion is sufficient to regulate *Srebf1* gene expression, as well as sterol response element (SRE)-mediated transcriptional targets (13, 21, 23, 71, 72). The SREBP-1 protein is also post-translationally activated by long-chain ceramides, which decrease INSIG-1 expression in an ER stress-dependent manner to promote SREBP-1 cleavage and maturation (21). Secondly, expression of fatty acid transporters (i.e. CD36, FABP, FATP2) and translocation of CD36 are regulated by ceramide (23, 73). CD36 translocation is thought to be mediated through ceramide activation of PKCζ (72). Lastly, direct regulation of genes involved in lipid metabolism by ceramide synthase enzymes has been reported. Notably, schlank, the drosophila CERS isoform, and mammalian CERS2 are capable of responding to intracellular fatty acid levels with direct binding and upregulation of *Srebf* expression and, conversely, downregulated expression of intracellular lipases (74, 75). Most of these actions have not been confirmed in the kidney; nonetheless, each could contribute to the lipotoxic effects of lipid accumulation observed with DKD in podocytes, TECs, and other kidney cell types (76–79).

### Insulin Resistance

Dysregulation of podocyte insulin signaling contributes to podocytopathy in DKD (80). Mice with podocyte-specific knockout of the insulin receptor or Akt2 develop characteristic DKD features including albuminuria, glomerular sclerosis, fibrosis, apoptosis, and mesangial matrix thickening (81, 82). These can occur in the absence of systemic hyperglycemia (81). Indeed, podocytes from *db/db* diabetic mice have decreased insulin-stimulated phosphorylation of Akt and are prone to apoptosis (83). The action of ceramide to inhibit Akt activation downstream of insulin signaling is well characterized (84, 85). Ceramides trigger an inhibitory phosphorylation of Akt by PKCζ, which prevents Akt/PKB translocation to the cell membrane (86–88). In addition, ceramide promotes dephosphorylation of Akt/PKB by PP2A to inhibit its activation (89–91). Aside from podocytes, insulin is a potent mediator of cellular function and survival in various kidney cell types and modulates important processes such as sodium reabsorption and gluconeogenesis in the proximal tubule (92). Systemically, ceramide-mediated insulin resistance within peripheral tissues (i.e. pancreas, liver, adipose, and muscle) can exacerbate chronic elevations in circulating glucose, inflammatory cytokines, and free fatty acids, all of which could add additional insult to the diabetic kidney (8, 93, 94). As such, ceramide’s antagonism of insulin signaling could induce a multitude of potential renal consequences.

### Mitochondrial Dysfunction and Impaired Fatty Acid Oxidation

DKD is characterized by impaired fatty acid oxidation and mitochondrial dynamics (56, 59). Patients with DKD have lower renal expression of fatty acid oxidation (FAO) genes (i.e. *CPT1a*, *CPT2*, *ACOX1*, *ACOX2*), as well as their transcriptional regulators *PPARa* and *PGC1a* (95). This phenomenon is also apparent in mouse models of DKD. Rescue of FAO in TECs *via* overexpression of PGC1α or treatment with the PPARα agonist fenofibrate prevents histopathological progression of nephropathy in mouse models of kidney fibrosis (95).

Several studies indicate that ceramides impair mitochondrial function in a wide range of experimental systems, including the kidney. Genetic and pharmacological ceramide-lowering interventions are sufficient to recover kidney PPARα expression (13, 42), although the mechanism is poorly understood. Several other studies have demonstrated that ceramides impair mitochondrial electron transport chain (ETC) activity. Hepatic mitochondrial complex inhibition was driven in *Cers2* haploinsufficient (96) and null (97) mice *via* accumulation of C16 ceramide. Accordingly, CERS6 overexpression in primary hepatocytes elicited the same effect (96), whereas CERS6 knockout prevented HFD-induced disruption of mitochondrial dynamics and function (98). Mechanistically, the CERS6-derived ceramides induce mitochondrial fragmentation by interacting with mitochondrial fission factor, which contributes to the dysfunction of the organelle in mouse models of metabolic disease (98).

### Apoptosis and Fibrosis

Kidney cell death and tissue fibrosis are common endpoints of the varying forms of CKD. Although DKD-specific studies are limited, the influence of ceramide in apoptosis is well defined (10, 99, 100). Indeed, ceramides elicit mitochondrial Bax-dependent apoptosis in cultured kidney cells in response to C(2)-ceramide or TNFα (67, 101, 102). Inhibition of oxidative stress in carbon tetrachloride-induced kidney fibrosis depleted kidney ceramide and prevented cell injury and death (103). Ceramides are also suspected to interact with and regulate TGF-β, a key regulator in fibrosis development (104–106). Recently, Dorotea et al. detailed a proposed mechanism for a positive feedback loop between SREBP1 and TGF-β, in which SREBP1 increases TGF-β expression and prevents exosomal degradation of the TGF-β receptor (70). This was demonstrated in transgenic mice overexpressing transcriptionally active SREBP-1a, which was sufficient to upregulate renal TGF-β expression and induce glomerulosclerosis and collagen deposition (79). As such, ceramides may indirectly activate TGF-β signaling and kidney fibrosis *via* their regulation of SREBP.

### Vascular Dysfunction

DKD is strongly associated with CVD, in part due to the prevalence of macro- and microvascular insults (107). Bioactivity of ceramide is thought to contribute to vascular dysfunction, both by inducing blood vessel vasoconstriction and increasing vascular permeability (108). Early studies demonstrated impairment of vasodilation in small blood vessels treated with C(2)-ceramide (109, 110), ceramide (109, 110), or palmitate (111), which corresponded with increased ROS production, decreased eNOS phosphorylation, and decreased NO. The Symons group confirmed that endothelial ceramide accumulation induces PP2A-eNOS co-localization, which prevents eNOS activation by Akt (111, 112). Additionally, accumulation of ceramide in human arterioles favors pathogenic H2O2-mediated flow-induced dilation (FID), rather than NO-mediated FID observed in healthy vessels (113). Inhibition of eNOS by ceramide, along with stimulation of mitochondrial ROS production (114), effectively decreases NO bioavailability and increases vascular susceptibility to sheer stress and damage (108). Ceramide also mediates disruptions to vascular integrity induced by inflammatory stimuli by disturbing endothelial tight junctions and inducing apoptosis (108, 115). Both systemic and local vascular impairments in vasodilation could exacerbate interglomerular pressure and injury (8, 108). Furthermore, in the DKD milieu of circulating glucose and inflammatory cytokines, increased permeability of the kidney endothelial barrier potentiates further damage.

### Alternative Mechanisms for Bioactive Ceramide Metabolites in DKD

Though our review has focused on ceramides, which have established roles in cellular stress, alternative bioactive sphingolipids could contribute to DKD. Within glomerular mesangial cells, elevated glucose or advanced glycation end products (AGEs) upregulate glycosphingolipid production (41) and/or sphingosine kinase activity (48) to promote mesangial proliferation and glomerular fibrosis. Beyond the general perceptions that S1P antagonizes ceramide as an anti-apoptotic, pro-survival signal (116, 117), its role in kidney disease is controversial. Several studies have supported a relationship between S1P, myofibroblast transdifferentiation, and ECM synthesis in kidney disease (reviewed in 118). Alternatively, loss of sphingosine kinase 1 activity led to poorer kidney outcomes in mouse models of DKD and kidney fibrosis (119, 120). Notably, S1P signaling outcomes are dependent upon downstream effectors of select G-protein-coupled S1P receptors, which are differentially expressed in diabetic kidneys (121). C1P has also been implicated as a regulator of mesangial cell proliferation (122) and podocytopathy (123). Lastly, gangliosides, namely GM3 and Gb3, accumulate in diabetic kidneys and may contribute to diabetic renal pathogenesis (124). Though we cannot cover these alternative sphingolipids in depth, we encourage the reader to further explore these alternative and compelling mechanisms, which have been reviewed elsewhere (118, 125–127).

## Conclusions

Thus far, the frontier of research delineating ceramides as bioactive drivers of DKD remains minimally explored. We present a unifying scaffold identifying ceramide as a potential central lipid mediator of DKD pathology. The scientific premise of the proposed action of ceramide in DKD is compelling. Additional work is needed, however, to demarcate the function of ceramide and sphingolipids in the onset of DKD and progression toward renal failure. Better use of kidney cell-specific animal models with genetic manipulation of ceramide accumulation and degradation will be useful to identify which cell types are most affected by the action of ceramide. Additionally, manipulation of ceramide synthases in the kidney will determine which ceramide species are most implicated in disease. The current natural history of DKD onset and advancement cannot explain the vast heterogeneity in DKD presentation and rate of kidney functional decline. We look forward to targeted mechanistic investigations of ceramide in DKD processes to improve the state of knowledge regarding DKD pathophysiology and to inform therapeutic development and treatment strategies.

## Author Contributions

RN, MP, and SS drafted the manuscript in collaboration with one another. RN generated the figures. All authors contributed to the article and approved the submitted version.

## Funding

The authors received research support from the National Institutes of Health (DK115824, DK116888, and DK116450 to SS), the Juvenile Diabetes Research Foundation (JDRF 3-SRA-2019-768-A-B to SS), the American Diabetes Association (to SS), the American Heart Association (to SS), and the Margolis Foundation (to SS).

## Conflict of Interest

SS is a consultant and shareholder with Centaurus Therapeutics.

The remaining authors declare that the research was conducted in the absence of any commercial or financial relationships that could be construed as a potential conflict of interest.
